# Examination of critical factors influencing ruminant disease dynamics in the Black Sea Basin

**DOI:** 10.3389/fvets.2023.1174560

**Published:** 2023-09-22

**Authors:** Margarida Arede, Daniel Beltrán-Alcrudo, Jeyhun Aliyev, Tengiz Chaligava, Ipek Keskin, Tigran Markosyan, Dmitry Morozov, Sarah Oste, Andrii Pavlenko, Mihai Ponea, Nicolae Starciuc, Anna Zdravkova, Eran Raizman, Jordi Casal, Alberto Allepuz

**Affiliations:** ^1^Departament de Sanitat i Anatomia Animals, Facultat de Veterinària, Universitat Autònoma de Barcelona, Barcelona, Spain; ^2^Food and Agriculture Organization of the United Nations (FAO), Regional Office for Europe and Central Asia, Budapest, Hungary; ^3^Food Safety Agency of the Republic of Azerbaijan, Baku, Azerbaijan; ^4^Veterinary Department, National Food Agency, Ministry of Environmental Protection and Agriculture of Georgia, Tbilisi, Georgia; ^5^Veterinary Control Central Research Institute, Ministry of Agriculture and Forestry, Ankara, Türkiye; ^6^Scientific Centre for Risk Assessment and Analysis in Food Safety Area, Ministry of Agriculture, Nubarashen, Yerevan, Armenia; ^7^Vitebsk State Academy of Veterinary Medicine, Vitebsk, Belarus; ^8^University Institute of Technology Nancy-Brabois, Lorraine University, Villers-lès-Nancy, France; ^9^National Sanitary Veterinary and Food Safety Authority, Bucharest, Romania; ^10^Faculty of Veterinary Medicine, State Agrarian University of Moldova, Chisinau, Moldova; ^11^Bulgarian Agency for Food Safety, Sofia, Bulgaria

**Keywords:** Black Sea, surveillance and control, ruminants, transboundary animal diseases, zoonoses

## Abstract

**Introduction:**

Ruminant production in the Black Sea basin (BSB) is critical for national economies and the subsistence of rural populations. Yet, zoonoses and transboundary animal diseases (TADs) are limiting and threatening the sector. To gain a more comprehensive understanding, this study characterizes key aspects of the ruminant sector in nine countries of the BSB, including Armenia, Azerbaijan, Belarus, Bulgaria, Georgia, Moldova, Romania, Türkiye, and Ukraine.

**Methods:**

We selected six priority ruminant diseases (anthrax, brucellosis, Crimean Congo haemorrhagic fever (CCHF), foot-and-mouth disease (FMD), lumpy skin disease (LSD), and peste des petits ruminants (PPR)) that are present or threaten to emerge in the region. Standardized questionnaires were completed by a network of focal points and supplemented with external sources. We examined country and ruminant-specific data such as demographics, economic importance, and value chains in each country. For disease-specific data, we analysed the sanitary status, management strategies, and temporal trends of the selected diseases.

**Results and discussion:**

The shift from a centrally planned to a market economy, following the collapse of the Soviet Union, restructured the ruminant sector. This sector played a critical role in rural livelihoods within the BSB. Yet, it faced significant challenges such as the low sustainability of pastoralism, technological limitations, and unregistered farms. Additionally, ruminant health was hindered by informal animal trade as a result of economic factors, insufficient support for the development of formal trade, and socio-cultural drivers. In the Caucasus and Türkiye, where diseases were present, improvements to ruminant health were driven by access to trading opportunities. Conversely, European countries, mostly disease-free, prioritized preventing disease incursion to avoid a high economic burden. While international initiatives for disease management are underway in the BSB, there is still a need for more effective local resource allocation and international partnerships to strengthen veterinary health capacity, protect animal health and improve ruminant production.

## 1. Introduction

Livestock production is critical for the subsistence of rural populations as a source of food, income, transportation, hides, and fertilizers, contributing to 40% of the agricultural economy worldwide (1). However, in recent decades, there has been a surge and spread of endemic and exotic diseases affecting livestock (2, 3), which significantly impact the sector and threaten public health and welfare (4). This surge has been intensified by several factors, including the high increase in international trade of animals and animal products (3), rise in intensive farming driven by higher market demands for animal protein and increasing middle-class purchasing power (5–7), changes in land use (8), shifts in migration and tourism patterns (9), and the effects of climate change (9).

TADs such as foot-and-mouth disease (FMD), lumpy skin disease (LSD), and peste des petits ruminants (PPR), along with zoonoses, particularly anthrax, brucellosis (*Brucella abortus* and *Brucella melitensis*), and Crimean Congo haemorrhagic fever (CCHF) (Table 1) are diseases that are either threatening ruminants or emerging in the Black Sea Basin (BSB). Ruminant production is the most important livestock subsector in most countries in the region, ensuring food security for rural populations and contributing significantly to national economies (16–25).

**Table 1:** Overview of the studied diseases.

		Disease	Agent	Main domestic host (s)	Transmission	Vaccine availability
Zoonoses	Bacterial	Anthrax (10)	*Bacillus anthracis*	All mammals	Contact with *B. anthracis* spores	Yes
		Brucella (11)	*Brucella abortus*	Cattle	Direct/indirect contact	Yes
			*Brucella melitensis*	Sheep and goats		
	Viral	CCHF (12)	CCHF virus g. Orthonairovirus f. Nairoviridae	Cattle, sheep, and goats	Tick-borne	No
TADs		FMD (13)	FMD virus g. Aphthovirus f. Picornaviridae	Cattle, sheep, goats, and swine	Direct/indirect contact	Yes
		LSD (14)	LSD virus g. Capripoxvirus f. Poxviridae	Cattle	Arthropod vector	Yes
		PPR (15)	Small ruminant morbilivirus g. Morbillivirus f. Paramixoviridae	Sheep and goats	Direct contact	Yes

g.: genus, f.: family.

Nevertheless, key aspects linked with the dynamics of these diseases in the region remain poorly understood. Knowledge gaps include disease geographic coverage and prevalence, morbidity and mortality rates, economic impact, and risk factors influencing their spread and persistence. These gaps arise from weaknesses in a country’s veterinary management programmes, which can be associated with lack of human resources (authorities, veterinarians and technicians) to sustain them, inadequate government funding for agriculture or livestock sectors, limited surveillance coverage (26), insufficient legislative action, and lack of support for implementing biosecurity measures (27). As a result, disease reporting is delayed, incomplete or biased, leading to ineffective responses to disease outbreaks (28, 29). These challenges are more pronounced in rural areas of lower to middle-income countries, as the BSB, where social inequality persists. In these regions, livestock, particularly ruminants, are ubiquitous and critical for livelihoods, and animal diseases hinder food security and the sector’s development.

This study characterizes ruminant production and its importance around the BSB and describes the disease status and management efforts (i.e., surveillance and control activities) for the selected ruminant diseases (Table 1) in nine countries of the region (i.e., Armenia, Azerbaijan, Belarus, Bulgaria, Georgia, Moldova, Romania, Türkiye, and Ukraine). It also explores the most relevant factors that may influence the incursion and spread of these diseases in the region.

## 2. Materials and methods

The current paper is a component of the GCP/GLO/074/USA project, which contributes to the broader “Global Framework for the Progressive Control of Transboundary Animal Diseases (GF-TADs)” initiative. This project targets nine countries located around the BSB, namely Armenia, Azerbaijan, Belarus, Bulgaria, Georgia, Moldova, Romania, Türkiye, and Ukraine. Herein, Armenia, Azerbaijan, and Georgia are referred to as “Caucasus,” when the statement is true for the three countries, and Türkiye is referred to as either “Thrace” or “Anatolia” when specific differences apply to each of the regions.

The primary focus of the project is on six diseases that are relevant for the region: anthrax, brucellosis, CCHF, FMD, LSD, and PPR. Consequently, this study focused on domestic ruminants (cattle, sheep and goats), which are the animal species most impacted by these diseases. These species are also interchangeably referred to as large ruminants (LR) and small ruminants (SR).

A report template was designed to collect information from each of the participating countries (Supplementary material S1). This document was developed by four authors of this paper (AA, DB-A, JC, and MA) as a semi-structured questionnaire. The selection of topics was based on the project’s objectives and aimed at addressing knowledge gaps in the BSB about the ruminant sector and the impact of the selected diseases. The initial version of the document was presented and shared with respondents from the nine participating countries during a virtual meeting. The final version of the report template accounted for edits and suggestions provided by the participants.

The report template was divided into two sections. The first section focused on the ruminant demographics, types of ruminant production, national and international trade, livestock markets, slaughterhouses, seasonal movements, and value chains. The second section focused on the six targeted diseases, requesting information on disease status, recent outbreaks, surveillance and control activities, awareness campaigns, and research activities in place.

Moreover, each report template requested information in two formats: narrative answers (e.g., description of a system or production type) and quantitative data in a database format (e.g., Excel datasheet). In some cases, quantitative data could complement descriptive information. To have high-quality figures, we requested the highest level of detail (e.g., the number of smallholder farms at the smallest administrative level) and, when applicable, exact locations (e.g., georeferenced locations of a livestock market). Further instructions prompted respondents to refer to additional documents like local veterinary authority national reports and national publications (i.e., grey literature).

One focal point (FP) of each participating country was appointed by FAO to answer the report template and collect country-specific information. FPs were carefully selected based on previous collaborations, the quality of their work, their expertise in the ruminant sector and selected diseases, and access to the data necessary for further analyses. FPs were based in each respective country and were working (or had recently worked) within relevant national institutions (e.g., veterinary services, food safety authorities, or national laboratories), during data collection. All nine FPs are co-authors of this paper.

FPs received the report template via email, filled it in with preliminary information, and iteratively and upon request, added further detail, following a back-and-forth exchange of emails and virtual meetings. Data collection was carried out by the FPs in collaboration with local peers, and all activities were coordinated with national authorities to request and obtain approval for data sharing. Data collection took place between October 2020 and December 2021.

Descriptive information and quantitative data were obtained and analysed from completed report templates. Then, data were assessed, and specific topics were selected to examine in this paper. To complement data on these topics, information was sourced from national reports and websites of the Food and Agriculture Organization of the United Nations (FAO), the World Organisation for Animal Health (WOAH), and the World Bank. To assess the economic importance of ruminant production for each country, we sourced data for the gross production value (GPV) of the main domestic production species from FAOSTAT (30). To find the proportional contribution of ruminant GPV to each country, we divided GPV for cattle, sheep, and goats, by the total GPV for all domestic species in 2020. Finally, ruminant distribution maps for ruminant populations (31–33) were sourced from FAO-NSAL (FAO’s Livestock Information, Sector Analysis and Policy) branch.

Quantitative data was managed, cleaned, harmonized, and collated in Microsoft Office Excel (2019), RStudio^®^ (34), and analysed and visualised in Quantum GIS (35) and RStudio^®^ (34).

## 3. Results

Selected topics from the nine participating countries were organized into two sections following the structure of the report template: (1) study region and ruminant-specific information, and (2) disease-specific information.

### 3.1. Study region and ruminant-specific information

#### 3.1.1. Study region

**Figure 1:**
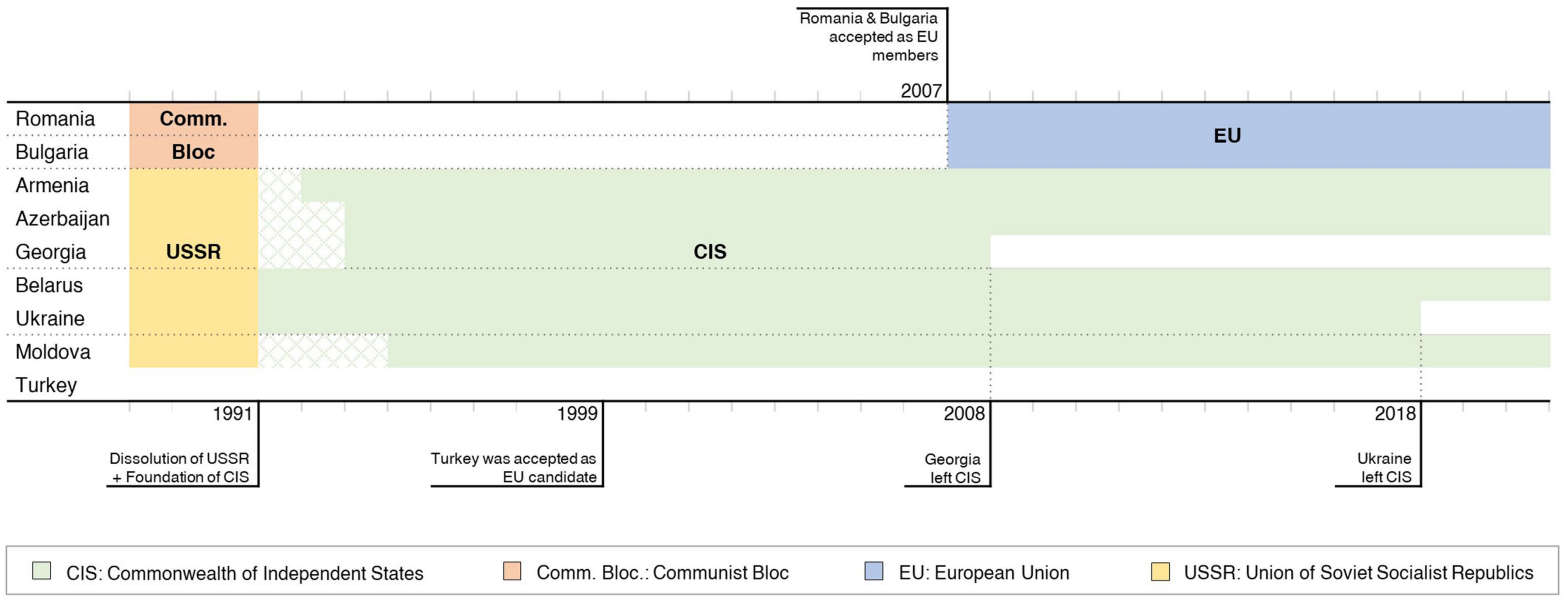
Political affiliations from 1988 to 2021 for the studied countries.

The main political changes and affiliations from countries of the study region between 1988 and 2021 are illustrated in Figure 1. Supplementary material S2 summarizes data for human and ruminant demographics, relevant economic indicators, and other characteristics of ruminant production. In 2020, most countries were classified as upper-middle-income economies, with the exceptions of Ukraine and Romania, which had a lower-middle-income economy and a high-income economy, respectively (36). The median GDP *per capita* of each region in 2020 was $4,547 USD, ranging from $3,725 USD in Ukraine to $12,896 USD in Romania. For livestock production indicators, Moldova and Bulgaria had the lowest contribution to agricultural GDP at 23%, while Belarus had the highest at 57% (30). The proportion of ruminant GPV (per total domestic production species) ranged between 23% in Moldova to 92% in Belarus (30). Further details about this indicator are supplied in Supplementary material S3.

#### 3.1.2. Ruminant demographics

**Figure 2:**
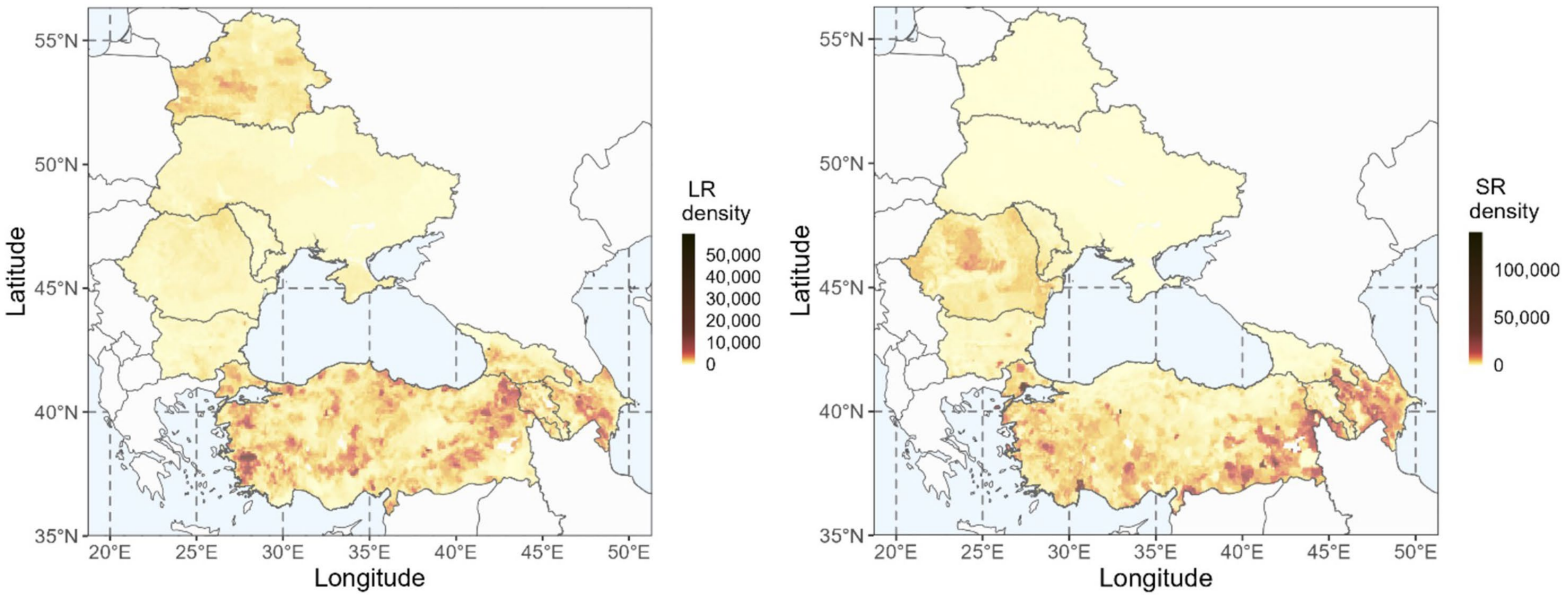
Distribution of large ruminants (LR) – cattle – and small ruminants (SR) – sheep and goats – in the study region. Source: GLW4 (Gridded Livestock of the World) data modified with countries’ data and adjusted for FAOSTAT 2020 (31–33).

The ruminant distribution varied significantly throughout the study region, both for LR and SR. LR heads ranged from 159,000 in Moldova to 18 million in Türkiye, whereas SR heads were lowest in Belarus (148,000) and highest in Türkiye (54 million). Figure 2 illustrates the spatial distribution for LR and SR in the region and shows higher abundance of LR in Belarus, certain regions of Türkiye, western Georgia, and Azerbaijan, and higher number of SR in parts of Türkiye (Thrace and southeast Anatolia), Romania, and Azerbaijan. Additionally, the figures highlight lower LR populations in Ukraine, Moldova, southern Romania, and northern Bulgaria, and lower SR populations in Belarus, Ukraine, Moldova, and northern Bulgaria.

#### 3.1.3. Production types

Countries classified ruminant production types using distinct terminology. To allow for comparisons, production types were grouped based on herd size and commercial purpose into smallholder and commercial farms, as defined in Supplementary material S6. In the Caucasus and Romania, over 90% of cattle farms were smallholdings, while Belarus had the highest proportion of cattle production in commercial herds. Across the entire region, more than 75% of herds keeping sheep and goats were smallholdings.

#### 3.1.4. Animal identification and registration systems

Most countries in the BSB had established National Animal Identification and Traceability Systems (NAITS). In contrast, the Caucasus had NAITSs under development, but not yet fully implemented at the time of data collection. In Azerbaijan, this system was being developed through a European Commission (EC) framework. It entered a regional pilot stage in late 2021 and began a country-wide phased implementation over 2022 (37). In Armenia, the Centre of Agribusiness and Rural Development (CARD), with support from the Austrian Development Agency (ADA) (38, 39), developed and conducted a pilot of its NAITS in the cattle sector in January 2022. Similarly, in Georgia, after a 5-year project supported by FAO and financed by the ADA and the Swiss Agency for Development and Cooperation (SDC) (40), the system was launched nationwide in February 2022 (40).

#### 3.1.5. National trade of live ruminants

Recordings of live animal movements were linked to the existence of a NAITS in each country. Therefore, most countries in the region recorded these movements within a national centralized database. Each registration included information regarding the individual identification of the animal and the farm of origin, the destination farm, and a veterinary health report issued by an official veterinarian.

Conversely, Georgia did not have a recording system for animal movements. In Armenia and Azerbaijan, movements between provinces were registered, but the record consisted solely of a paper-based veterinary health certificate. These records were issued by official veterinarians and archived in regional divisions. There were no centralised databases for recording live animal movements in these three countries.

Live animal movements were characterized by a seasonal pattern that is not detailed in this paper. Nevertheless, it is worth noting that these movements significantly surged during cultural-religious celebrations such as Novruz, Kurban Bayram, and Ramadan Bayram in Azerbaijan and Türkiye, in which animals are transported to cities to be ritually slaughtered. Similarly, Easter and St George’s Day in Bulgaria and Romania were also preceded by an increase in live animal movement due to the traditional consumption of mutton.

Furthermore, it is important to highlight that livestock trade was closely linked to animal density in each region, the demand for animal protein in densely populated areas, the location of slaughterhouses, and specific commercial partnerships with regions or countries. For example, in Georgia, ruminant trade primarily occurred from west to east due to the high exports to Azerbaijan. As for Bulgaria, the southern regions, where LR and SR production was more intense, also had an increased movement of ruminants. Moreover, in Türkiye, ruminants were moved from small to large provinces, and more specifically from east to west and north to south of the country.

##### 3.1.5.1. Livestock markets

The role of livestock markets in live ruminant trade varied across the region. Azerbaijan and Türkiye run ten and 150 licensed live animal markets, respectively, which played a significant role in ruminant trade. During Kurban Bayram in these two countries, markets worked exceptionally to sustain the surge in animal movements. In Armenia, Georgia, and Bulgaria, these facilities existed but were not as relevant for animal trade. In Belarus, official markets for live ruminant trade were absent, instead occasional fairs and exhibitions were held at the district level and on a small scale. In the same country, ruminant trade for breeding purposes occurred through state breeding companies. In Ukraine, smallholders used live animal markets for local ruminant trade.

##### 3.1.5.2. Seasonal movements

Pastoralism includes seasonal movements to pastures and can be sub-classified as nomadism, transhumance, or agropastoralism (definitions provided in Supplementary material S6). These practices are key to the seasonal sourcing of graze and water for livestock and were common across the study region. In Bulgaria, the Caucasus, Romania, and Türkiye, transhumant animals were moved to summer pastures, often found in mountainous areas, in spring and summer, and to lowland pastures or stables in autumn and winter. Migrating months had slight variations yearly depending on weather and pasture conditions. Georgia, Azerbaijan, and Türkiye set up Veterinary Surveillance Points (VSP) along migration routes. These premises primarily focused on mass vaccination campaigns in Azerbaijan, but also served as rest points for supplying feed and water, as sanitary checkpoints for health status control, and anti-parasitic application in Georgia and Türkiye. The mingling of animals from various herds, regions or even neighbouring countries was common in seasonal pastures. Consequently, these animals were vaccinated either before going to pasture or during migration in VSPs. Furthermore, movements to seasonal pastures were recorded in centralized systems for movement control in Bulgaria, Romania (41) and Türkiye; however, these recordings, similarly to national movements, were not done in the Caucasus.

In Belarus, Moldova, and Ukraine, ruminants kept in smallholdings or smaller private farms in rural settings grazed seasonally in fields surrounding their holdings, in an agropastoral manner.

#### 3.1.6. International trade of live ruminants

Partner trading countries with the BSB region are presented in the last two columns of Supplementary material S2. International trade of live animals was done based on country partnerships, contingent on the trust in the exporting country’s animal health capacity and/or the sanitary status for the main contagious zoonoses and TADs (at a specific time) (42). To guarantee disease freedom on entry into a country, imported live ruminants were accompanied by a health certificate validated by a veterinarian of the exporting country’s competent authority. Particularly for the importation of live animals (and animal products) into the EU, the intra-EU trade, and EU exports of live animals, TRACES (Trade Control and Expert System) (43), an EC online platform, facilitates sanitary certification required for trade and centralizes trade information. Thus, Bulgaria and Romania along with other BSB countries exporting live animals or animal products into the EU, used this platform.

Similar to national live animal movements, international trade was influenced by cultural-religious events. Therefore, a surge in live animal imports preceded Kurban Bayram and Ramadan Bayram in Azerbaijan and Türkiye, and Easter and St George’s Day in Bulgaria and Romania.

### 3.2. Disease-specific information

#### 3.2.1. Disease status, surveillance, and control activities

**Figure 3:**
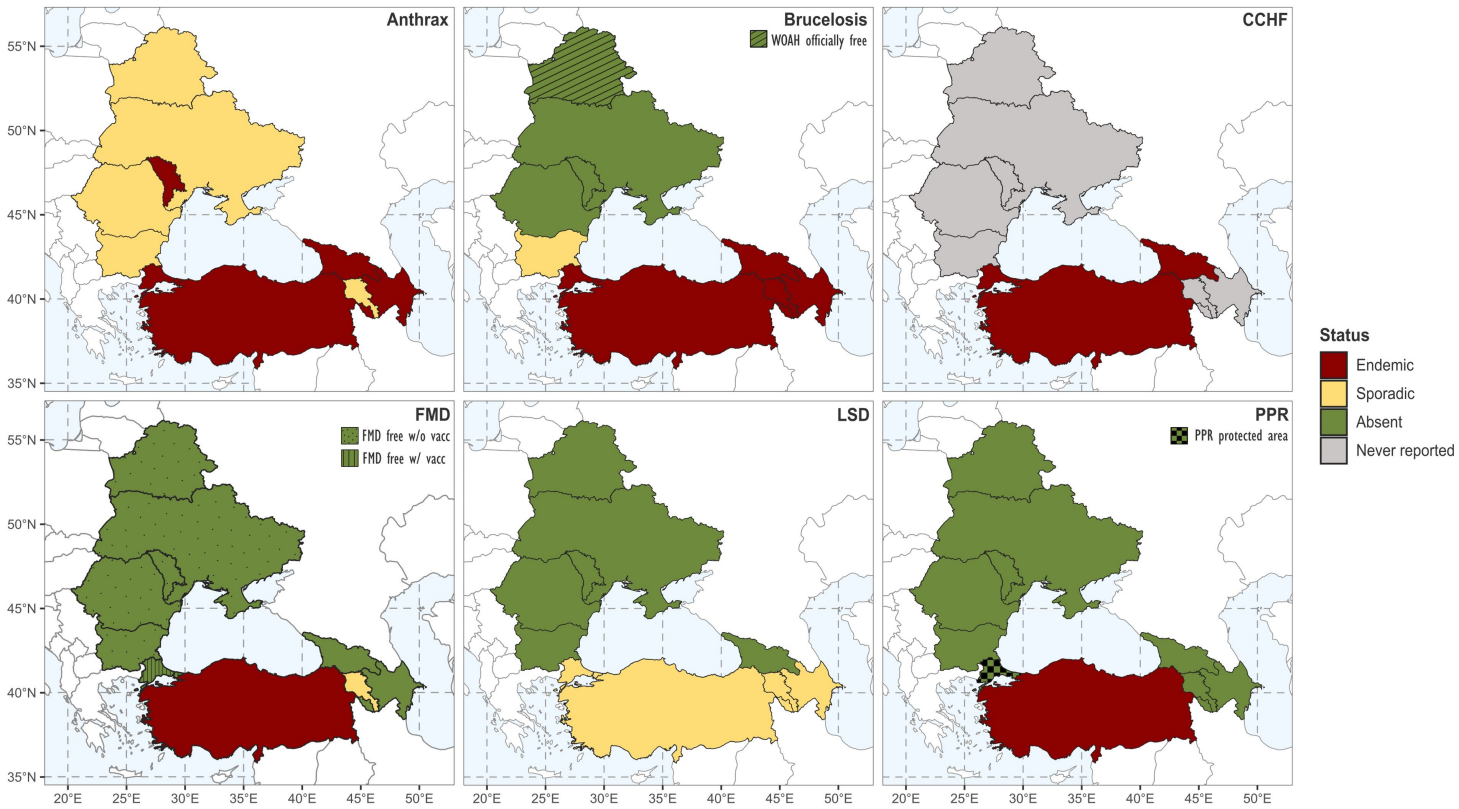
Status of target diseases in the study region. Brucellosis status refer to *Brucella abortus* and *Brucella melitensis*.

Figure 3 illustrates the country-level disease statuses for each selected disease. Countries self-classified their disease status as endemic, sporadic, or absent (definitions in Supplementary material S6). An *Absent* status was subclassified for brucellosis as “officially free” and for FMD as “officially free with or without vaccination” when WOAH officially recognised these disease statuses.

In Supplementary material S4, a table summarizes key details for the six studied diseases in each of the countries of the BSB. Moreover, temporal trends of disease outbreaks per country from 2010 to 2020 are shown in Supplementary material S5. In this subsection of the results, we review the disease status and management practices applied in the region.

##### 3.2.1.1. Anthrax

Anthrax was endemic or sporadic in all countries. All countries implemented passive surveillance and, upon suspicion, applied further clinical examinations, sampling, and testing. Due to the environmental nature of this disease, most national management programmes, in addition to guidelines for disease containment and carcass disposal, also included regulations for historically infected fields (e.g., signalling, fencing, digging restrictions, and awareness campaigns). Vaccination was compulsory for all ruminants in Azerbaijan, and Moldova, and all LR in Armenia. A risk-based vaccination approach was applied for all ruminants in Bulgaria, Georgia, Romania, Türkiye, and Ukraine, and exclusively for SR kept or moved to high-risk areas in Armenia. In Belarus, anthrax vaccination was not conducted.

##### 3.2.1.2. Brucellosis

Brucellosis was endemic in the Caucasus and Türkiye, sporadic in Bulgaria, and absent in all other countries of the BSB. In 2012, Belarus was officially recognised by WOAH as brucellosis-free, and to maintain this status, serosurveillance was conducted every three years. Moreover, surveillance was exclusively passive for Georgia, and active and risk-based in all other countries. In most of the BSB, passive surveillance for brucellosis was associated with the report and investigation of abortions in ruminants, which is a syndrome of this disease, but not exclusive to *Brucella* spp. infection. In Azerbaijan, Georgia, and Türkiye, vaccination for brucellosis was mandatory for all ruminants and performed at the same time as serosurveillance. Brucellosis vaccination was not part of the national veterinary control plan in Belarus, Bulgaria, Moldova, Ukraine, or Romania.

##### 3.2.1.3. Crimean Congo haemorrhagic fever

CCHF was endemic in Georgia and Türkiye. These countries applied control measures upon outbreak identification, focusing on tick control and community awareness campaigns. These activities comprised the application of acaricide sprays to ruminants, including during seasonal migrations from early spring to late autumn, and environmental tick elimination. Educational campaigns in Türkiye promoted contact restriction between livestock and wildlife, and tick management. These campaigns were included in the state budget at no cost to farmers. For the remaining countries, CCHF had never been reported in ruminants and there was no national surveillance programme in place. At the time of data collection, no licensed vaccine was available for CCHF in ruminants.

##### 3.2.1.4. Foot-and-mouth disease

The WOAH official FMD status varied between the two regions of Türkiye: Anatolia was classified as endemic, and Thrace held FMD-free status with vaccination. FMD was sporadic in Armenia, absent in Georgia and Azerbaijan, while in all other countries, WOAH recognised the official status FMD-free without vaccination.

FMD surveillance was active in the Caucasus and Türkiye, as well as in regions of Bulgaria and Romania. The countries of the Caucasus were collaborating with EuFMD through the Progressive Control Pathway for Foot and Mouth Disease (PCP-FMD) to design and establish risk-based surveillance programmes. As part of these efforts, they implemented NSP (Non-Structural Protein) and SP (Structural Protein) serosurveys to evaluate the FMD virus circulation, seroconversion, and vaccination coverage. In regions bordering Thrace, Bulgaria conducted risk-based serosurveys on a sample of ruminants every three months. While in Romania, surveillance focused on clinical examination of LR and SR on high-density premises (e.g., live animal markets, exhibitions, ports, and airports), serosurveillance of all ruminants close to international borders, and SR upon their arrival from seasonal pastures.

In Türkiye, the FMD management programme in 2021 aimed to achieve FMD-free status without vaccination in Thrace and FMD-free status with vaccination in Anatolia by 2025 (44). In Thrace, control measures comprised suspect FMD case culling, restrictions on live animal imports from Anatolia, and strict adherence to sanitary legislation. In Anatolia’s southeast provinces bordering FMD-endemic countries, surveillance activities were enhanced and risk-based. Moreover, in case of an FMD outbreak, Türkiye conducted a field investigation, and vaccination, established a *cordon sanitaire*, animal quarantine, and thorough cleaning and disinfection, organized training, and awareness campaigns, and closely monitored all premises within a 10 km radius of the event.

FMD vaccination varied throughout the BSB. Türkiye vaccinated LR twice a year, and SR once a year only in Thrace. In case of an outbreak in Anatolia, SR were also vaccinated in established protection and surveillance zones. In Azerbaijan, LR were vaccinated twice a year (spring and autumn) and SR once a year, while Armenia, applied the same strategy only in high-risk areas. Since 2017, Georgia has conducted vaccination exclusively in high-risk areas, based on risk assessments, which considered seasonal migration, international borders with FMD-endemic countries, live animal markets, and informal trade.

##### 3.2.1.5. Lumpy skin disease

LSD was sporadic in Armenia, Azerbaijan, and Türkiye, and absent in all other countries. Surveillance activities varied: clinical examination was conducted in Belarus to a sample of LR in spring and summer, and in the six regions of Bulgaria bordering Thrace monthly. Georgia had active participatory surveillance, Türkiye implemented both active and passive surveillance activities, and all other countries only applied passive surveillance. Compulsory vaccination was practised nationwide in Azerbaijan, Bulgaria, and Türkiye, and in high-risk areas of Armenia and Georgia. Vaccination was not applied in Belarus, Moldova, Romania, or Ukraine.

##### 3.2.1.6. Peste des petits ruminants

PPR was endemic in Türkiye and absent in all other countries. In March 2021, Thrace was granted the classification of “PPR-protected area.” PPR surveillance varied across the BSB: Belarus did not conduct it, Moldova, Ukraine, and Anatolia exclusively applied passive surveillance, while Thrace and all other countries applied active surveillance. In Bulgaria, areas previously affected by PPR (2018 outbreak) implemented enhanced surveillance, and regions bordering Thrace applied risk-based serosurveillance on a sample of SR every two months. In Romania, active surveillance included clinical inspection of a sample of SR herds before and after pasture season.

Vaccination was implemented in Georgia, following the first PPR occurrence in 2016. In Türkiye vaccination was conducted, yet it ceased in Thrace after the region was granted a “PPR-protected area” classification in March 2021. This measure, coupled with strict live SR movement restrictions from Anatolia to Thrace, aimed at Thrace’s application for WOAH PPR zonal freedom status in 2023. In Anatolia, PPR vaccines were applied to all newborn SR and unvaccinated adults.

## 4. Discussion

In this paper, we summarized the ruminant production sector and reviewed the sanitary status and management of six diseases affecting ruminants (anthrax, brucellosis, CCHF, FMD, LSD, and PPR) in the BSB. Furthermore, we explored key factors contributing to the introduction and spread of these diseases in the region.

### 4.1. Post-Soviet Union reform

The fall of the Soviet Union caused a deterioration of public infrastructures and services across the former Soviet Union (FSU) and Communist Bloc countries, significantly affecting agricultural and livestock sectors (18, 45–48). In BSB countries, except Belarus, changes included the shift from collective and state-owned farms to private ownership, removal of government subsidies to the livestock sector (49), closure of large slaughterhouses (50), and depletion in resource allocation to veterinary services (51). Such factors left livestock production in the hands of unspecialized farmers, and unsupervised by veterinary services (52), resulting in increased disease incidence (52, 53). Thereafter, the region suffered a steep decline in the number of ruminants (49, 54) and, in some countries, as Ukraine and Belarus, a significant abandonment of agricultural lands (55). These abrupt structural changes were followed by a transition phase with gradual agricultural recovery and increasing productivity (56). Yet, rural poverty, particularly in the Caucasus and Moldova, persists and requires new and efficient policy measures that enable technological development and access to market channels and services (51). EU’s farmer association model could aid smallholders of the FSU to actively engage to improve their marketing, input supplies, and support services (57).

### 4.2. Rural livelihoods and pastoralism

Pastoralism played a critical role in rural areas in most countries of the BSB (16–18, 20–25, 46, 58–60), creating a unique interdependence between ruminants, farmers, and the environment (61, 62). Preserving this practice is crucial, given its resilience to severe climates in arid and inhospitable areas, socio-cultural importance, and the potential opportunities brought to younger generations (62). However, its sustainability in the BSB is a matter of concern. Ageing rural farmers show reluctance to adopt new technologies and measures to improve animal production and health, and the mass migration of younger populations to urban centres leaves families without essential support for farming activities (57). Moreover, they have limited access to veterinary services also caused by ageing rural veterinarians, and difficulties in attracting young graduates due to low-income prospects and prevailing urban migration trends (57). These factors result in underperforming veterinary services (63) and high costs for disease management impeding improvements, even when advancements are made at higher levels (28). Solutions for these challenges need to be explored, as building private veterinary capacity and developing training programmes for veterinary paraprofessionals.

In addition, initiatives addressing pastoralism’s limited sustainability and its associated risks to ruminant health and welfare are underway (64, 65). In Georgia (62), Türkiye, and Azerbaijan, VSPs were established along migration routes. In Armenia, the “Project Coordination Platform for Sustainable Management of Natural Grazing Lands – Pastures and Grasslands” was launched to address pasture management-related problems in the country (66). Internationally, the Pastoralist Knowledge Hub (PKH) by FAO aids the development of synergies for dialogue and pastoralist development, while an extension of the Performance of Veterinary Services (PVS) (67) evaluation tool prioritizes finding solutions to control animal diseases in pastoralist areas (63). Collectively, these initiatives aim to foster and protect pastoralism while ensuring its sustainability.

### 4.3. Disease management and related factors

Disease management in the Caucasus and Türkiye was often inefficient. Nonetheless, improvements were being made in the field. Particularly in the Caucasus, the full operability of the NAITSs is expected to make disease management programmes (68) and disease traceability (69) more efficient. As a result, these improvements will positively influence animal health and ruminant production, ultimately, leading to better trade opportunities and economic growth in these countries.

Sociocultural-religious events in Türkiye and Azerbaijan prompted the implementation of contingency plans and extraordinary measures, which, at times, proved inefficient in preventing disease introduction and spread. In fact, the epidemiological investigation conducted upon the PPR incursion to Bulgaria in July 2018 concluded that the high demand and resulting price difference of mutton between Bulgaria and Thrace during these festivals contributed to increased informal movements of people and animals (70).

In the BSB, only Türkiye reported the presence of all studied diseases. This can be attributed to its unique conditions, including a large ruminant population, vast geographical area with socio-economic disparities, and extensive rural regions. Moreover, its shared borders with six countries including Syria and Iraq, where social unrest leads to informal movement of people with their livestock, create a significant pathway for disease spread (71, 72). Recognising the high risk to animal and public health through this route, Türkiye introduced legislative acts for border control and supervision of the main roads (73, 74). These acts are open to amendment, and they aim to manage and identify informal/illegal trade for livestock and products of animal origin, along with enforcing animal culling. Following the implementation of these controls, a national report highlighted a significant reduction of nearly 95% and 50% of confiscated smuggled animals and animal products, respectively, caught during border controls conducted in 2011 and 2018. This demonstrates the successful impact of these actions (75).

EU countries, such as Bulgaria and Romania, had high resource allocation for disease management and prioritised the prevention of disease incursion to reduce economic losses. These countries followed harmonized live animal trade regulations set by the EC, enforcing additional control measures and trade restrictions (76) in the event of an exotic disease incursion. Therefore, responses to Bulgaria’s FMD (2011), LSD (2016), and PPR (2018) outbreaks were quick and intensive (77). And LSD and FMD outbreaks resulted in an economic burden estimated at €8 million (78), and $1.5 billion USD annually (79), respectively. To prevent disease re-emergence and further economic losses, disease management activities established upon these events, were still in place as of 2021.

Moreover, Thrace’s proximity to Europe and shared borders with the EU through Bulgaria and Greece, prompted the establishment of partnership programmes between the EU and Türkiye. These initiatives involved significant investments to curb disease introduction and spread into Europe, while also promoting trade opportunities (80, 81). Therefore, FMD and PPR statuses varied between Thrace and Anatolia, leading to distinct classifications by WOAH, along with distinct approaches for disease management and movement control in these two regions.

### 4.4. The exception of CCHF

Türkiye and Georgia were the only countries in the BSB reporting the presence of CCHF in ruminants. In spite of this, past studies identified CCHF virological or serological evidence and the presence of competent vectors in most countries of the study region, except for Belarus (12, 82–84), while CCHF human cases were also notified in Bulgaria and Türkiye (84). Non-reporting of CCHF in ruminants was linked to two factors. Firstly, its exclusion from national veterinary programmes resulted in the absence of routine official surveys, and secondly, the subclinical nature of the disease in these species allows it to circulate unnoticed (85–87). Nevertheless, domestic ruminants play an important role in the epidemiology of the disease as they are involved in its vector life cycle (83) and amplification and spread of the virus (88, 89). Moreover, ruminant CCHFV antibody titers correlate with virus presence in a region (84), as well as human disease incidence (82). Given CCHF’s public health threat, including potential human incurred deaths, the prudent course of action is to include the disease in national veterinary programmes. This would ensure regular disease monitoring and prompt response to any reported cases.

### 4.5. Current initiatives

Achieving effective disease management requires not only efficient resource allocation for national disease preparedness and response but also promoting collaborations with other countries and unions. An initiative that strengthens regional alliances for TADs management is the GF-TADs, a joint FAO and WOAH effort, created to support capacity building and the establishment of disease management programmes based on regional priorities (77). GF-TADs’ priority diseases in the BSB include brucellosis, FMD, LSD, and PPR. Under this initiative, the Global Strategy for the Control and Eradication of PPR aims to control and eradicate PPR and strengthen veterinary services (90, 91). Additionally, FAO’s PCP-FMD guides endemic countries in progressively managing FMD risks and reducing its impacts and viral circulation (92–94).

### 4.6. Limitations

Study limitations were linked to country-specific factors and data quality issues. Absent or not fully operable NAITSs are likely to have affected data validity, and completeness is limited due to unregistered herds along with underreporting across the BSB. Underreporting is often linked to farmers’ poor disease awareness, distrust in governmental authorities, risk of penalty or stigmatization, or at a higher level, lack of capacity to enforce regulations (95) and low transparency. Additionally, variability in data availability and spatial resolutions between countries led to reduced accuracy of certain indicators or made it impossible to compare and examine others. Finally, data quality might have been affected by resource reallocation during the COVID-19 pandemic, which partly coincided with the two-year data collection period.

### 4.7. The armed conflict in Ukraine

The armed conflict in Ukraine, starting in February 2022, had a significant impact on its livestock sector. Since its beginning, the conflict led to decreased agricultural production due to land abandonment, animal losses from death or forced slaughtering, and reduced demand for meat and milk due to mass emigration (96). It has disrupted the accessibility to veterinary services, vaccines, medication (97), and critical inputs, such as feed and fodder (96), compromising disease prevention and control, and increasing the risk of stress, malnourishment, and susceptibility to disease in livestock. Moreover, amongst security issues, unavailability of consumables and equipment, and competing urgent priorities, appropriate carcase disposal became challenging. These effects are expected to reshape ruminant demographics, its associated production sector, and value chains, particularly in front-line regions. International cooperation is vital to address the consequences on livestock health and revive the sector post-conflict. Guidelines aiming to support the livelihoods of livestock-keeping communities in humanitarian emergencies that affect livestock are in place (98, 99), being used to alleviate the consequences of the presented conflict.

## 5. Conclusion

This study provides a comprehensive overview of the ruminant production sector and the management of six major diseases of concern in the BSB. By examining the effects of the post-soviet reform, the importance of pastoralism, differences in disease management and countries’ response to disease incursion, as well as the influence of cultural events and political affiliations on live animal trade, we have gained a valuable understanding of how these different factors work together to determine disease dynamics in the region.

Unlike the other studied diseases, CCHF was not included in veterinary management plans, and not surveyed in ruminants across the region, presenting a public health threat. Furthermore, the armed conflict in Ukraine starting after data collection will likely have a significant impact on ruminant production and animal disease emergence in this country, with potential spread to neighbouring countries.

Finally, despite recent developments in veterinary infrastructures, including the implementation of NAITSs in the Caucasus, substantial support from international agencies and targeted initiatives for ruminant disease management, the need to improve animal health persists, particularly in rural and remote regions. A thorough understanding of the primary challenges, needs, and constraints faced by smallholders in each specific country context is essential. Establishing priorities and closely assessing them in collaboration with farmers, national stakeholders, and international agencies, will aid in identifying opportunities for more effective disease management strategies contributing to alleviating and preventing future outbreak scenarios. These considerations go hand in hand with providing incentives for rural development, by seeking financial aid, efficiently allocating financial and human resources, and most importantly ensuring the sustainability of the implemented strategies.

## Data availability statement

The datasets presented in this article are not readily available for public access due to restrictions imposed by the governmental entities of the nine participating countries. The data that support the findings of this study were used under license from these entities and are subject to confidentiality and legal constraints. Requests to access the datasets should be directed to margarida.decastro@uab.cat.

## Ethics statement

Ethical review and approval was not required for the study of animals in accordance with the local legislation and institutional requirements.

## Author contributions

AA, DB-A, JC, and MA developed the report template and contributed to the conception and design of the study. JA, TC, IK, TM, DM, AP, MP, NS, and AZ were hired as country FPs to fill in the report template and provide quantitative data and descriptive information for the project. SO wrote a document summarizing preliminary information. MA performed the data management, descriptive analysis and visualization materials, and wrote the first draft of the manuscript. All authors contributed to the article and approved the submitted version.

## Funding

The project or effort depicted was or is sponsored by the United States Department of Defense, Defense Threat Reduction Agency. The content of the information does not necessarily reflect the position or the policy of the Federal Government of the United States, and no official endorsement should be inferred.

## Conflict of interest

The authors declare that the research was conducted in the absence of any commercial or financial relationships that could be construed as a potential conflict of interest.

## Publisher’s note

All claims expressed in this article are solely those of the authors and do not necessarily represent those of their affiliated organizations, or those of the publisher, the editors and the reviewers. Any product that may be evaluated in this article, or claim that may be made by its manufacturer, is not guaranteed or endorsed by the publisher.
