# Corrigendum: Examination of critical factors influencing ruminant disease dynamics in the Black Sea Basin

**DOI:** 10.3389/fvets.2024.1376956

**Published:** 2024-02-28

**Authors:** Margarida Arede, Daniel Beltrán-Alcrudo, Jeyhun Aliyev, Tengiz Chaligava, Ipek Keskin, Tigran Markosyan, Dmitry Morozov, Sarah Oste, Andrii Pavlenko, Mihai Ponea, Nicolae Starciuc, Anna Zdravkova, Eran Raizman, Jordi Casal, Alberto Allepuz

**Affiliations:** ^1^Departament de Sanitat i Anatomia Animals, Facultat de Veterinària, Universitat Autònoma de Barcelona, Barcelona, Spain; ^2^Food and Agriculture Organization of the United Nations (FAO), Regional Office for Europe and Central Asia, Budapest, Hungary; ^3^Food Safety Agency of the Republic of Azerbaijan, Baku, Azerbaijan; ^4^Veterinary Department, National Food Agency, Ministry of Environmental Protection and Agriculture of Georgia, Tbilisi, Georgia; ^5^Veterinary Control Central Research Institute, Ministry of Agriculture and Forestry, Ankara, Türkiye; ^6^Scientific Centre for Risk Assessment and Analysis in Food Safety Area, Ministry of Agriculture, Nubarashen, Yerevan, Armenia; ^7^Vitebsk State Academy of Veterinary Medicine, Vitebsk, Belarus; ^8^University Institute of Technology Nancy-Brabois, Lorraine University, Villers-lès-Nancy, France; ^9^National Sanitary Veterinary and Food Safety Authority, Bucharest, Romania; ^10^Faculty of Veterinary Medicine, State Agrarian University of Moldova, Chisinau, Moldova; ^11^Bulgarian Agency for Food Safety, Sofia, Bulgaria

**Keywords:** Black Sea, surveillance and control, ruminants, transboundary animal diseases, zoonoses

In the original article there was an error in the **Funding** statement. The incorrect statement was used. (“The project GCP/GLO/074/USA “Global Framework for the Progressive Control of Transboundary Animal Diseases (GF-TADs)” was funded by the United States Department of Defense, Defense Threat Reduction Agency (DTRA). The content of the information does not necessarily reflect the position or the policy of the Federal Government of the United States, and no official endorsement should be inferred.”)

The correct Funding statement appears below:

