# Supplementary material for: Examination of critical factors influencing ruminant disease dynamics in the Black Sea Basin
Source: Front Vet Sci. 2023 Sep 22;10:1174560. doi: 10.3389/fvets.2023.1174560 (PMC10557248; doi:10.3389/fvets.2023.1174560)
Supplement: Supplementary file 1 [file Data_Sheet_1.pdf]

S1: Report template

Name			
Date		Country	
Name of contributors (add more rows if necessary)		Email of contributors	

This report template has the objective of gathering information about the national ruminant production sector, and six ruminant diseases - anthrax, brucellosis, Crimean Congo haemorrhagic fever (CCHF), foot-and-mouth disease (FMD), lumpy skin disease (LSD) and peste des petits ruminants (PPR) - including the related risk factors, surveillance, and control strategies.

Please follow the structure provided below to write the report, replying to each question in detail, and referencing complementary documents, which do not need to be translated at this stage.

These complementary documents should be attached as appendices. They may include:

- Internal industry reports.
- Veterinary Service or Laboratory reports.
- National databases, e.g.: outbreaks, surveillance results, livestock numbers, etc.
- National agricultural magazine's articles.
- National scientific publications.
- Maps/ Graphs.
- Master or PhD thesis (documents not published in international journals).

Note:

a. If the source document is written in a language other than English, indicate it with reference to the question and add a brief summary of the information it contains and the language, so we can decide if it needs translation.

b. Scientific publications in international journals (in English) do not need to be included, since they will be covered by FAO research partner.

RUMINANT SPECIFIC INFORMATION

1. Include data at the maximum resolution level (i.e. individual farm or the lowest administrative level available). Please note that the classification provided is generic, but may be different in your country. Please provide additional categories (e.g. mixed purpose, dairy goats or sheep, extensive systems, nomadic herds, etc. to reflect the existing categories within your country).
    - Refer to an Excel file

2. Provide an outline or narrative of the main commercial and/or backyard ruminant production systems in the country and their characteristics, e.g. definition of each farm type (how is a backyard, commercial or semi-commercial defined), how do they operate in rough terms, proportion of farms with mixed species, etc.

3. Did the proportion of ruminant farms/ animals change in the last 5 years? Describe how.

4. Provide an outline of the value chains for ruminant production in the country (i.e. who is involved and what is the contribution and the profit of the participants). If available, provide also major documents/studies about this topic.

5. Animal trade markets: describe where live animals are traded, how often, and under what conditions.

6. **Animal movements**

   6.1. Are movements between farms recorded and available in a database? If so, please describe the data fields captured and the level of detail. Please describe the rough amount and type of movements that are not recorded.

   6.2. Are there seasonal animal movements, e.g. animals that go to summer pastures or large nomadic/transhumance movements? If yes, are these movements recorded in a database and available? If so, please describe the data fields captured and the level of detail. If not, describe the movement season and pattern (i.e. frequency and location), as well as the approximate number of animals involved.

7. **Trade**

   7.1. For each ruminant species, describe imports and exports of the following products, including countries involved and quantities:

      7.1.1. Live animals

      7.1.2. Animal products

         7.1.2.1. Meat

         7.1.2.2. Milk and dairy products

         7.1.2.3. Semen and embryos

         7.1.2.4. Hides

   7.2. Main trade routes within the country. Describe the geographical aspect of trade routes and transport media for the domestic market, as well as the actors involved, e.g. existence of middlemen, live animal markets or agricultural fairs, animals are sent straight to slaughterhouses, etc.

   7.3. Has illegal animal trade been identified within the country and with other countries? Describe in what instances this happens and what are the most difficult borders to control.

DISEASE-SPECIFIC INFORMATION

Disease	Endemic / Sporadic / Absent	If absent or sporadic, state the date of the last introduction	Is the disease reportable?
Anthrax			
Brucellosis			
CCHF			
FMD			
LSD			
PPR			

8. **For each specified disease**

8.1. Provide the database for the outbreaks and/or surveillance results existent in the country in the last 10 years (if available one row per affected farm), if possible, with the following information:

8.1.1. Date

8.1.2. Location (or region)

8.1.3. Geocoordinates (if available)

8.1.4. Species (i.e. cattle, goat or sheep) plus production system if available, e.g. dairy, beef, extensive, etc.

8.1.5. Number of animals present on the farm.

8.1.6. Number of animals affected.

8.1.7. Number of dead animals.

8.1.8. List other fields captured in the national database.

8.2. Provide the database of the cases in humans in the last 10 years for anthrax, brucellosis, and CCHF, if possible, with the following information:

8.2.1. Disease (anthrax, brucellosis, or CCHF)

8.2.2. Date

8.2.3. Location

8.2.4. List other fields captured in the national database.

8.3. For each disease, if there is no outbreak database, please describe the outbreaks that occurred in the last 10 years:

8.3.1. Where did it occur in the country?

8.3.2. What was the source of the introduction of disease into the country?

8.3.3. How did it spread?

8.3.4. How many animals were affected/ killed?

8.3.5. Economic impact?

8.3.6. How was it controlled?

9. **Surveillance Plans**

9.1. Are National Surveillance Programmes implemented? If yes, describe the surveillance program for each of the diseases.

10. **Control measures**

10.1. Is there a national vaccination plan for the different diseases or any other sort of control plan? If yes, please describe.

10.2. Is it common practice the application of insecticides/repellents/acaricides on ruminants to control ticks? (Possible vectors of LSD and CCHF)

10.3. Are there any distribution maps and info about relevant vectors for CCHF, i.e. Hyalomma ticks? If yes, please describe.

10.4. Are there any wild animals known or suspected to be responsible for the spreading or being reservoirs of any of these diseases in the country? If yes, please describe.

11. **Disease Awareness**

11.1. Do authorities (or others) organize training sessions, seminars, brochures, and leaflets to inform/prevent (about) the referred diseases? Indicate for which diseases this has been done and describe briefly the awareness actions implemented.

11.2. Are there any biosecurity improvement programs?

12. **University/ NGOs/ Government research of disease**

12.1. Indicate if there is any national research for any of the six diseases in agricultural/ veterinary schools/ NGOs. If yes, could you provide a contact person and/ or any publication/ report referring to it? Please include their contact details (i.e. email) and area of expertise.

S2: Demographics, economic indicators, and ruminant production indicators in the study region.

Country	Human population (2020)[2]	GDP/capita (2020-S$)[3]	% Livestock production/ agricultural GDP (2020)[1]	% GPV ruminants/ GPV domestic species (2020)[1]	LR population*		LR production types (%)*		SR population*		SR production types (%)*		Animal identification and traceability system	International trade (live animals)	
					Head (Thous.)	% LR in region	Smallholder farms	Commercial farms	Head (Thous.)	% SR in region	Smallholder farms	Commercial farms		Exports to	Imports from
Armenia	2 963 234	4 266	46	76	600	1.8	95	5	690	3	95	5	No (under development)	GEO, Middle East	EU, KAZ, RF, UKR
Azerbaijan	10 093 121	4 221	43	78	2 484	7.5	91	9	8 189	10.2	82	18	No (under development)	GEO, TK, UAE	BLR, EU, GEO, KAZ, RF, TK, UKR
Belarus	9 379 952	6 424	57	92	4.300	13.1	2	98	148	0.2	91	9	Yes	KAZ, UZB	EU<br>SR: low imports
Bulgaria	6 934 015	10 079	23	48	722	2.2	60	40	2 015	2.5	76	24	Yes	Balkans, Caucasus, TK	EU
Georgia	3 722 716	4 267	38	65	721	2.2	95.5	4.5	685	0.9	95	5	No (under development)	ARM, AZE, Iran, Middle East	ARM, AZE, BLR, EU, RF, UKR
Moldova	2 620 495	4 547	23	23	159	0.5	74	16	845	1.1	87	13	Yes	AZE, Middle East	BLR, CH, EU, KAZ, RF, Serbia, TK, UKR, UK
Romania	19 257 520	12 896	31	46	1 867	8.8	95	5	12 541	15.6	96	4	Yes	EU, Middle East[4]	EU[4]
Türkiye	84 339 067	8 536	29	76	18 158	55.1	80[5]	20[5]	54 113	67.3	93	7	Yes	AZE, Cyprus, Iraq, Middle East, PK	AZE, EU, South America
Ukraine	44 132 049	3 725	24	80	2 900	8.8	66	44	1 144	1.4	87	13	Yes	ARM, AZE, EU, KAZ, MDV, Middle East, TK, UZB	EU

* Ruminant census values refer to 2021. GPV: Gross production value, LR: large ruminants, SR: small ruminants, Thous.: Thousand

S3: Proportion of gross production value (GPV) per production species (cattle, goats, poultry, sheep, swine) in each studied country, in 2020.

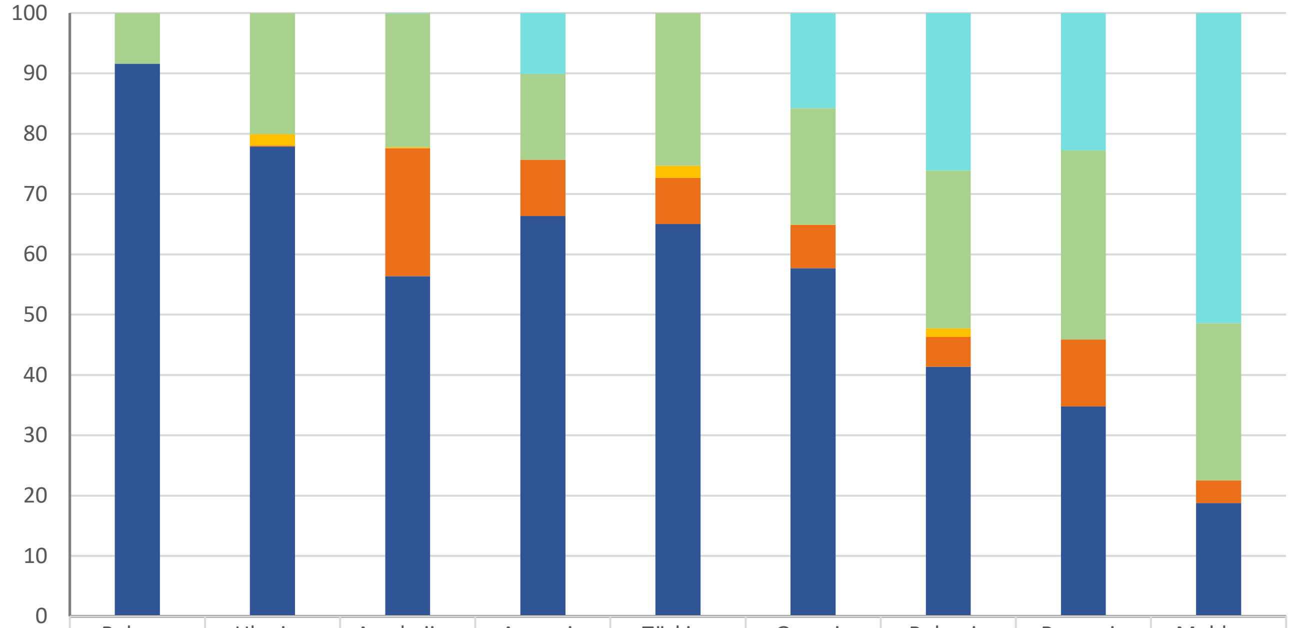

	Belarus	Ukraine	Azerbaijan	Armenia	Türkiye	Georgia	Bulgaria	Romania	Moldova
Swine	0.0	0.0	0.1	10.1	0.0	15.7	26.1	22.8	51.4
Poultry	8.4	20.1	22.2	14.2	25.3	19.3	26.2	31.4	26.1
Goats	0.0	1.9	0.1	0.0	2.0	0.0	1.4	0.0	0.0
Sheep	0.0	0.1	21.3	9.3	7.6	7.2	4.9	11.1	3.7
Cattle	91.6	77.9	56.4	66.4	65.0	57.7	41.3	34.8	18.8

Cattle Sheep Goats Poultry Swine

*Source FAOSTAT[1]

S4: Overview of disease status, surveillance activities, and vaccination in the study region in 2021, for domestic large ruminants (LR) and small ruminants (SR).

Country / Disease	Armenia	Azerbaijan	Belarus	Bulgaria	Georgia	Moldova	Romania	Türkiye	Ukraine
**Anthrax**	Armenia	Azerbaijan	Belarus	Bulgaria	Georgia	Moldova	Romania	Türkiye	Ukraine
Status	Sporadic	Endemic	Sporadic	Sporadic	Endemic	Endemic	Sporadic	Endemic	Sporadic
Last outbreak	2019	-	[1]*	2018	-	2016	2020	-	2017
Surveillance	Passive	Passive	Passive	Passive	Passive	Passive	Passive	Passive	Passive
Vaccination	Yes<br>LR: young 2x/ yr., adult: 1x/ yr.<br>SR: risk-based 1x/ yr.	Yes<br>LR & SR<br>1st year: 2x/yr.<br>Adults: 1x/yr.	No	Yes (risk-based)<br>All SR & LR kept or moved to areas w/ 1 (or +) past outbreaks in last 50 yrs.: 1x or 2x/yr.	Yes (risk-based)<br>Susceptible LR & SR in high-risk areas	Yes (All)	Yes (risk-based)	Yes (risk-based)<br>Susceptible animals before seasonal movements	Yes (risk-based)<br>areas w/outbreaks in <5yrs: all LR & SR (2x/yr.), stalling/ grazing >5yrs: adult LR & SR - 1x/yr., young LR & SR - 2x/yr.
**Brucellosis**	Armenia	Azerbaijan	Belarus	Bulgaria	Georgia	Moldova	Romania	Türkiye	Ukraine
Status	Endemic	Endemic	Absent<br>WOAH official free status	Sporadic	Endemic	Absent	Absent	Endemic	Absent
Last outbreak	-	-	XX century	2019	-	1995	1965	-	[2]*
Surveillance	Active<br>Roz-Bengal (risk-based): ELISA/CFT confirmation<br>All LR & SR: 2x/yr. (spring and autumn)	Active<br>ELISA and Roz-Bengal<br>Sample of 50% of LR and 20% of SR.	Active<br>Every 3 yrs. to maintain the official free status	Active, passive<br>LR: non-dairy: 1x/yr. >24m 2x/yr. BTM; SR: 1x/yr. for SR >6m based on herd size	Passive (2018–present)	Active, passive<br>ELISA and Roz-Bengal	Active, passive<br>All LR: BTM >3x/yr. ea.3m. or blood sample 1x/yr. [3;12]m; SR: 1x/yr.	Active, passive	Active<br>LR & SR 1x/yr for all adult animals
Vaccination	No	Yes (REV1 and S19)<br>LR: S19 to all females 3-8m and all non-pregnant females.<br>SR: REV1 all female 3-8m and all non-pregnant female[6]	No	No	Yes<br>Started in 2019	No	No	Yes (all female LR and SR)<br>LR: first dose 3–6m; second dose: 4 to 12m after the 1st;<br>SR: 3–6m-old female lambs and kids and breeding male animals	No
**CCHF**	Armenia	Azerbaijan	Belarus	Bulgaria	Georgia	Moldova	Romania	Türkiye	Ukraine
Status	Absent	Absent	Absent	Absent	Endemic	Absent	Absent	Endemic	Absent
Last outbreak	Never reported	Never reported	Never reported	Never reported	-	Never reported	Never reported	-	Never reported
Surveillance	No surveillance	No surveillance	No surveillance	No surveillance	Passive<br>Non-notifiable disease	No surveillance	No surveillance	Passive<br>Non-notifiable disease	No surveillance
Vaccination	Not available	Not available	Not available	Not available	Not available	Not available	Not available	Not available	Not available

FMD	Armenia	Azerbaijan	Belarus	Bulgaria	Georgia	Moldova	Romania	Türkiye	Ukraine
Status	Sporadic	Absent (of clinical cases)<br>No WOAH official status	Absent<br>Free without vaccination<br>(WOAH official status)	Absent<br>Free without vaccination<br>(WOAH official status)	Absent (of clinical cases)<br>No WOAH official status	Absent<br>Free without vaccination<br>(WOAH official status)	Absent<br>Free without vaccination<br>(WOAH official status)	Anatolia region Endemic,<br>Thrace (Free zone w/ vaccination since 2010)<br>Thrace has the WOAH official status	Absent<br>Free without vaccination<br>(WOAH official status)
Last outbreak	2016	2001	1982	2011	2002[7]	1979	Never reported	-	1988
Surveillance	Active<br>Risk-based, (NSP, SP)<br>1x/yr. post-vaccination spring/autumn	Active<br>Risk-based (NSP, SP)<br>1x/yr. post-vaccination spring/autumn	Passive	Active in 6 southern regions bordering Thrace, tests every 3m.<br>Passive in whole country	Active<br>Risk-based (NSP, SP)	Active	Active, passive<br>Clinical exam. & serosurveillance in high-risk areas	Active<br>Clinical exam. since 2013<br>Thrace(only): serosurveillance	Active
Vaccination	Yes<br>High-risk areas<br>LR: 2x/yr.<br>SR: 1x/yr.	Yes<br>LR: 2x/yr. spring-autumn<br>SR: 1x/yr. spring/autumn	No	No	Yes (Risk-based since 2017)<br>All susceptible LR& SR in high-risk areas)	No	No	Yes<br>LR: 2x/yr. spring-autumn<br>SR: only Thrace, 1x/yr. 21d pre-seasonal pastures	No
**LSD**	Armenia	Azerbaijan	Belarus	Bulgaria	Georgia	Moldova	Romania	Türkiye	Ukraine
Status	Sporadic	Sporadic	Absent	Absent	Absent	Absent	Absent	Sporadic	Absent
Last outbreak	2016	2014	Never reported	2016	2018	Never reported	Never reported	2021	Never reported
Surveillance	Passive	Passive	Active (clinical examination)<br>Passive	Active: regions bordering Thrace, clinical exam.<br>Passive: whole country	Active, passive	Passive	Passive	Active, passive	Passive
Vaccination (Only LR)	Yes<br>Adult cattle: high-risk zones (borders with neighbouring countries)	Yes	No	Yes<br>2016: blanket vaccination<br>Last 5 yrs.: vaccination coverage 84% to 98%	Yes<br>Risk-based	No	No	Yes<br>>3m whole country	No
**PPR**	Armenia	Azerbaijan	Belarus	Bulgaria	Georgia	Moldova	Romania	Türkiye	Ukraine
Status	Absent	Absent	Absent	Absent	Absent	Absent	Absent	Anatolia: Endemic<br>Thrace: PPR-protected area	Absent
Last outbreak	Never reported	Never reported	Never reported	2018	2016	Never reported	Never reported		Never reported
Surveillance (Only SR)	Active<br>Risk-based<br>(ELISA and PCR in 2019/2020 w/ no positive results)	Active<br>ELISA	No programme	Active & risk-based (regions bordering Thrace): samples every 2m; Enhanced in regions w/ PPR in 2018<br>Passive: whole country	Active	Passive	Active, passive<br>Clinical exam. before & after pasture season	Passive	Passive
Vaccination	No	No	No	No	Yes	No	No	Thrace: No (since March 2021); Anatolia: >3m; unvaccinated adults + all SR in outbreak areas	No

LR: large ruminants, SR: small ruminants, yr.: year; m: month; d: day, BTM: Bulk tank milk. [1]*last reported case affected equine (2019); [2]*last reported case affected swine (2008).

S5: Temporal distribution from 2010 to 2021 of outbreaks in large ruminants (LR) and small ruminants (SR) for the selected diseases by country.

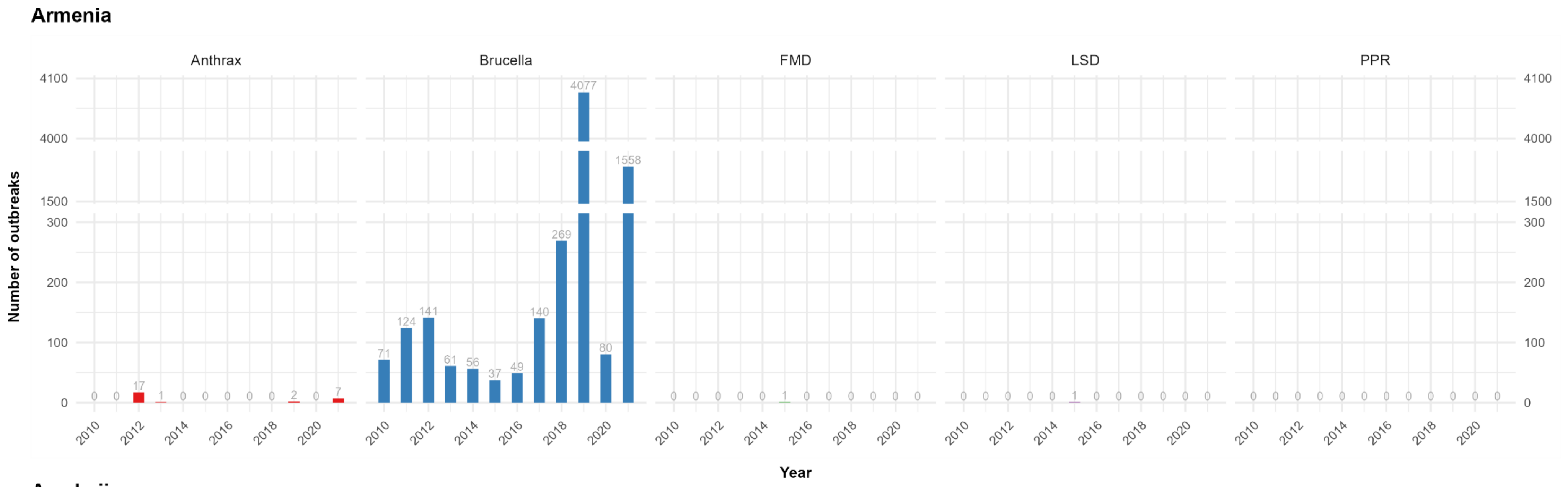

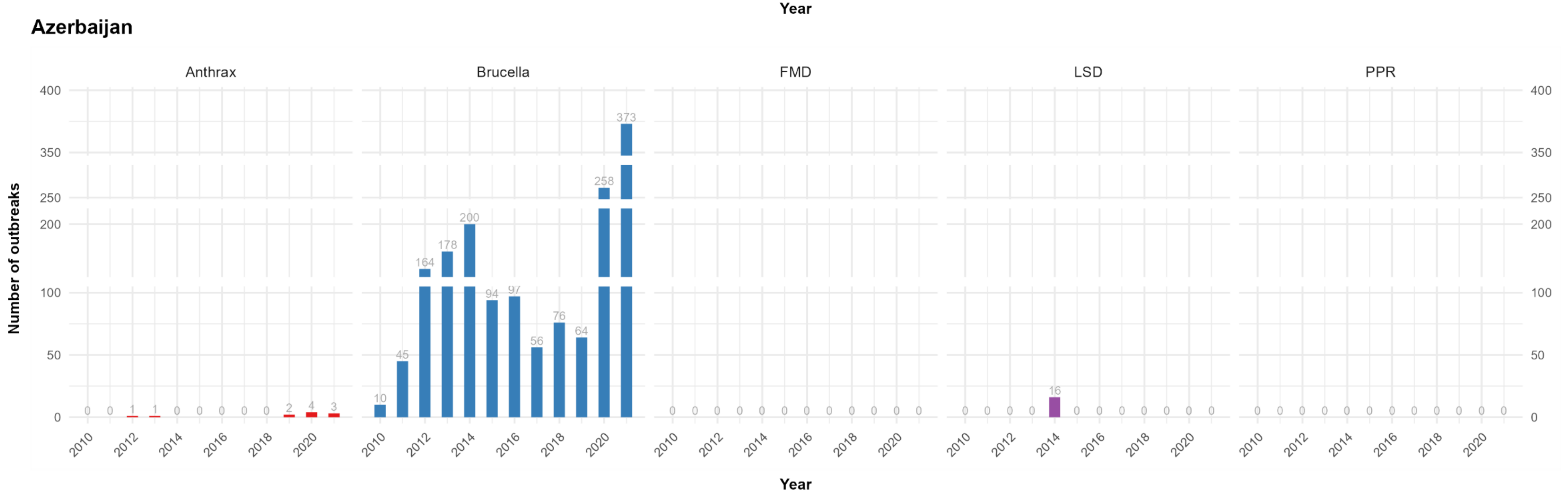

Belarus

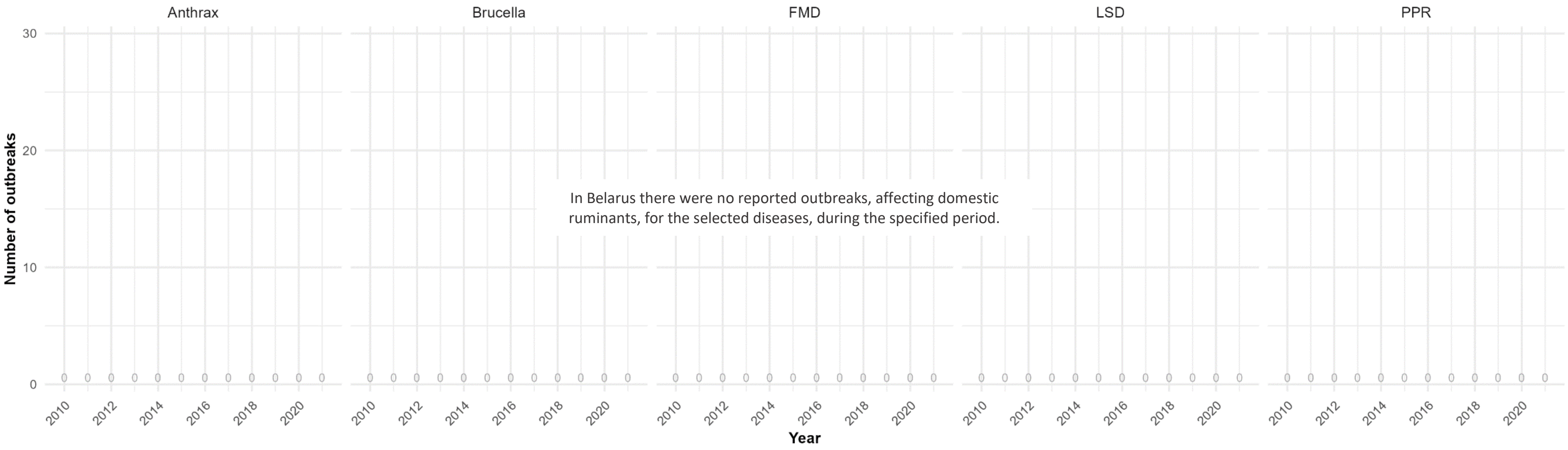

Bulgaria

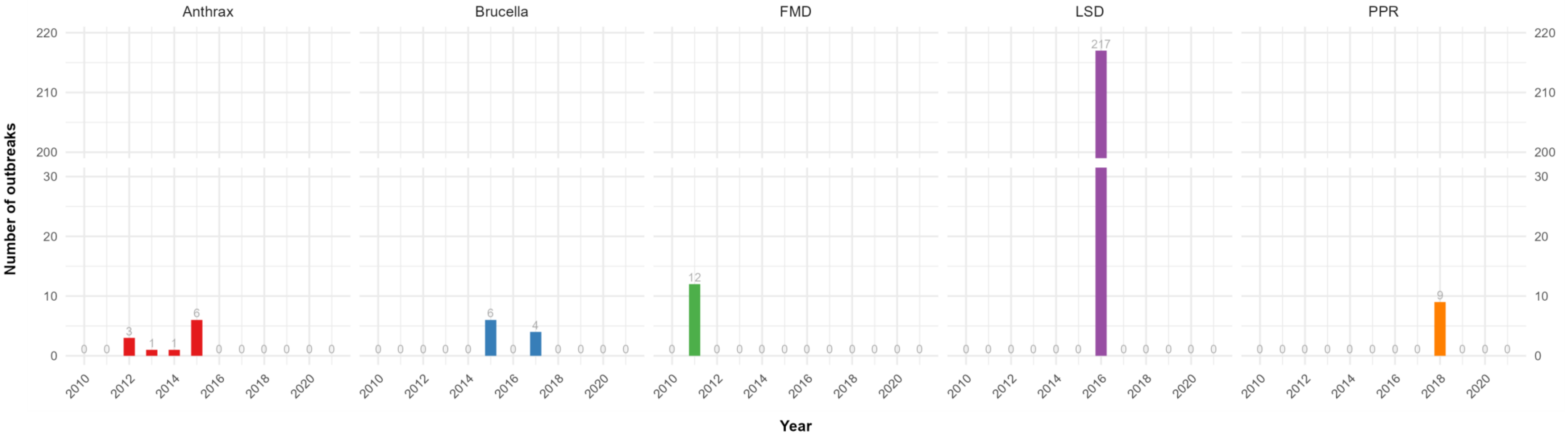

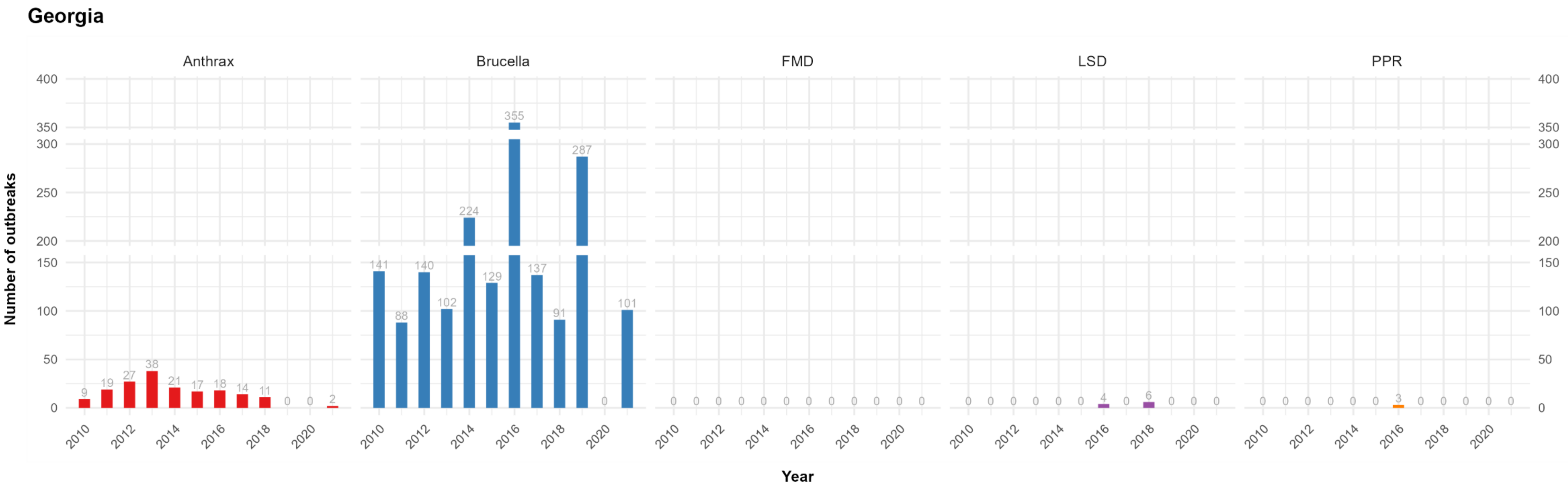
Georgia
Anthrax
Brucella
FMD
LSD
PPR
Number of outbreaks
Year
9
19
27
38
21
17
18
14
11
2
141
88
140
102
224
129
355
137
91
287
101
4
6
3

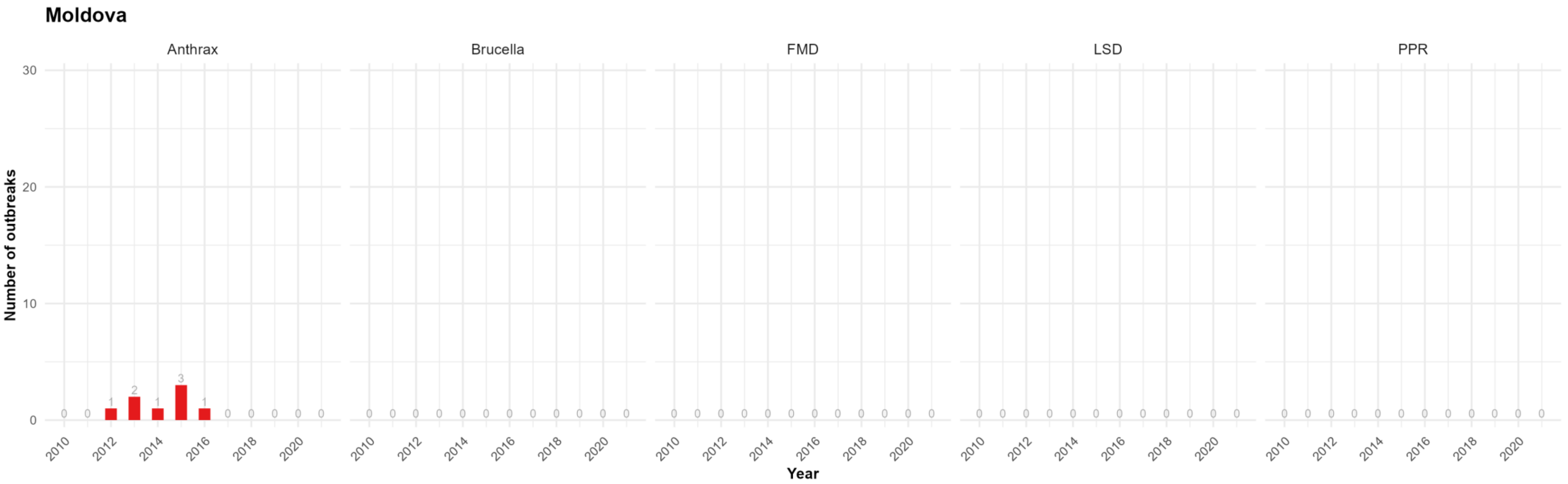
Moldova
Anthrax
Brucella
FMD
LSD
PPR
Number of outbreaks
Year
1
2
1
3
1

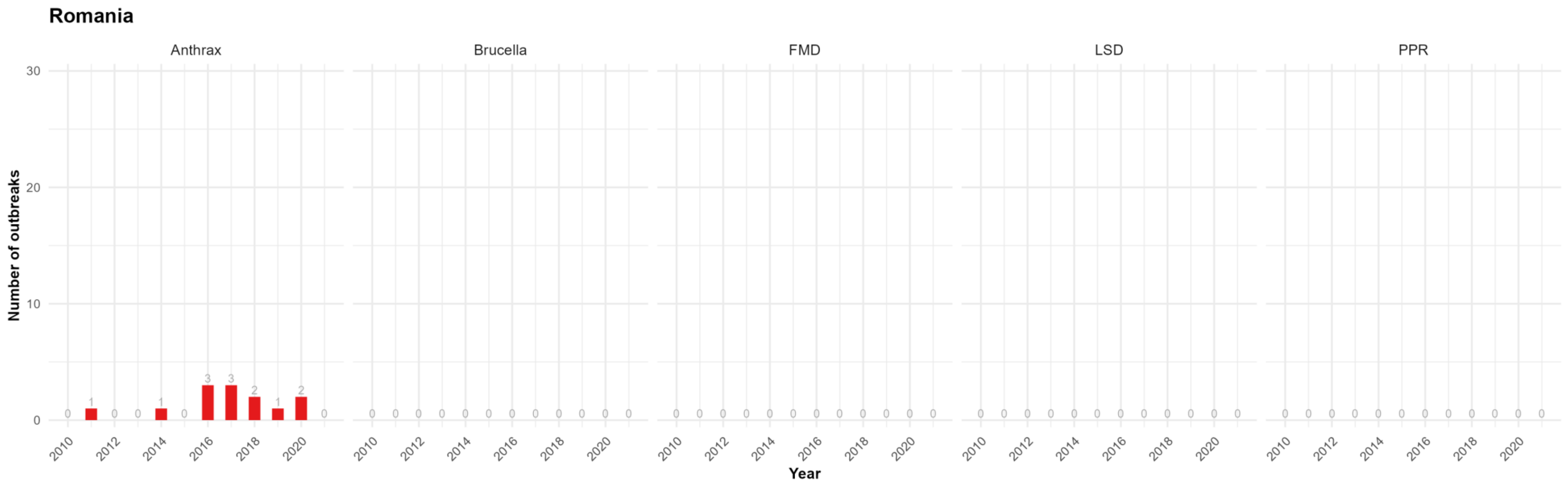
Romania
Anthrax
Brucella
FMD
LSD
PPR
Number of outbreaks
Year

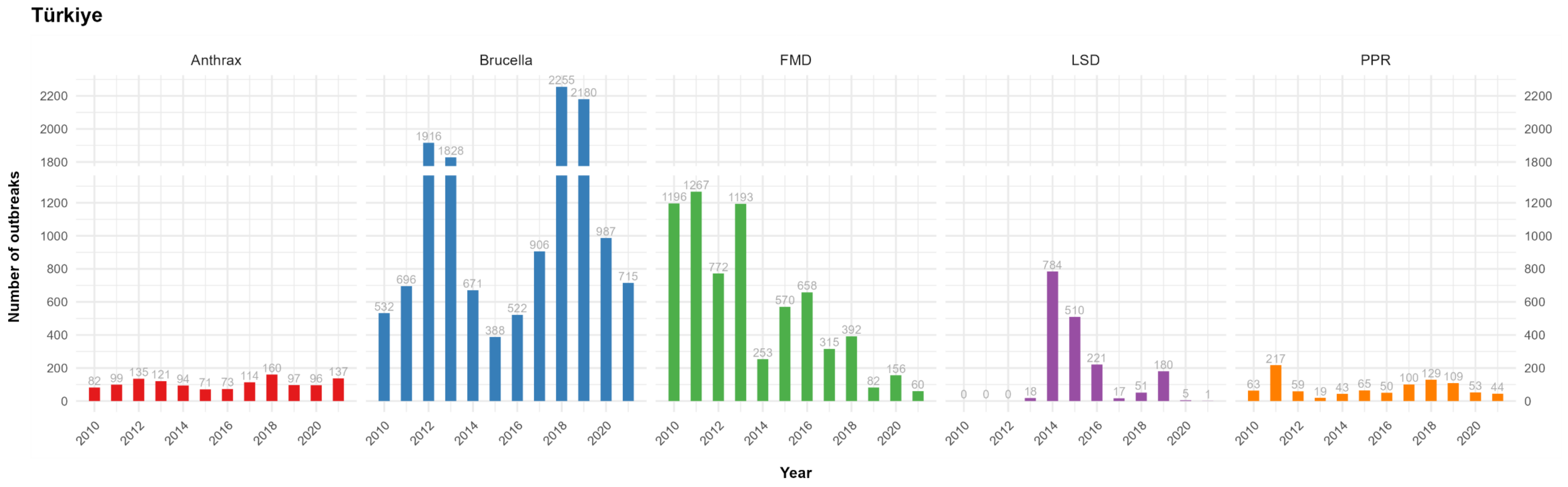
Türkiye
Anthrax
Brucella
FMD
LSD
PPR
Number of outbreaks
Year

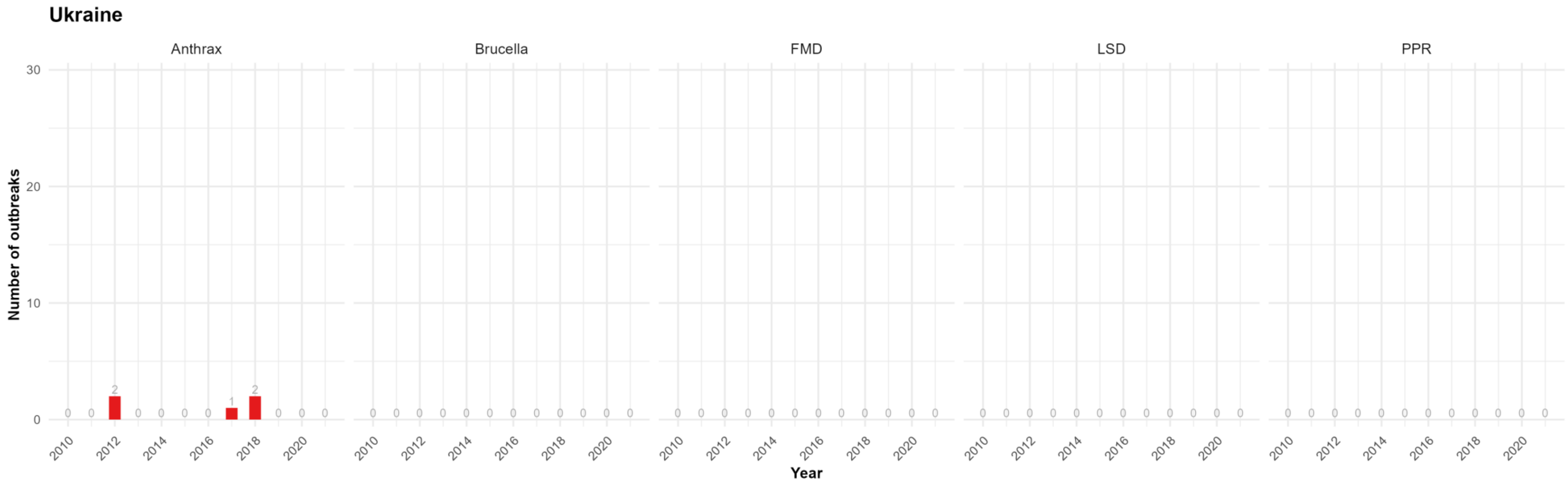
Ukraine
Anthrax
Brucella
FMD
LSD
PPR
Number of outbreaks
Year
0
10
20
30
2010
2012
2014
2016
2018
2020
2
1
2

S6: Definitions

Farm type classification:

- **Smallholder farms**, interchangeably called family or backyard farms, include a range of producers, from the most impoverished to those that gradually become involved with markets at a local or national level[8]. This term is related to a holding with a smaller size (lower number of animal heads), often characterized by having fewer resources and low productivity, and its main purpose is subsistence or semi-subsistence.
- **Commercial farms** are defined as having a larger scale and a higher number of animals. They are associated with a high investment, for their use of modern technology and wider access to resources, present higher efficiency, and have the main purpose of commercializing final products.

Pastoralism is an extensive livestock production practised in drylands and characterised by seasonal movements and common use of natural resources[9].

- **Nomadism** is based on the flexible seasonal migration of livestock that rarely has a home base.
- **Transhumance** is the regular movement of herd animals between fixed points to utilize pasture and water seasonal availability. It can be vertical when movements are based on ancient routes to mountainous regions; or horizontal, based on opportunistic movements developed over a few years, in disrupted areas due to climatic, political, and/or economic changes.
- **Agropastoralism** is the mixed production of crops and livestock that are grazed close to their village[10].

Disease Status

- **Sporadic disease:** is a disease that occurs infrequently and irregularly in space and time[11,12].
- **Endemic disease:** is the presence of a disease or infectious agent over a long time in a population within a geographical area[11,12].
- **Transboundary animal diseases (TADs),** as the key feature that defines them, are diseases that can spread rapidly between countries and reach epidemic proportions, leading to significant socioeconomic impacts in a region[13,14]. Moreover, effectively managing and controlling these diseases requires constant cooperation between countries[14]. Which justifies constant cooperation between countries for effective disease management and control[14].
- **Zoonoses:** are naturally transmissible diseases between animals and humans[15], that raise additional concerns for public health, in addition to their impact on livestock.

References

1. FAO. FAOSTAT. Published 2022. Accessed July 5, 2022. https://www.fao.org/faostat/en/#data/QV
2. World Bank Open Data. Population, total - Armenia, Azerbaijan, Turkiye, Moldova, Romania, Belarus, Ukraine, Georgia, Bulgaria | Data. Published 2022. Accessed November 3, 2022. https://data.worldbank.org/indicator/SP.POP.TOTL?end=2020&locations=AM-AZ-TR-MD-RO-BY-UA-GE-BG&most_recent_year_desc=true&start=1960
3. World Bank Open Data. GDP per capita (current US$) - Armenia, Azerbaijan, Georgia, Belarus, Bulgaria, Moldova, Romania, Ukraine, Turkey | Data. Published 2022. Accessed March 24, 2022. https://data.worldbank.org/indicator/NY.GDP.PCAP.CD?locations=AM-AZ-GE-BY-BG-MD-RO-UA-TR-ES-PT
4. Grodea M, Ionel I. The Romanian export with livestocks-live animals: A far-reaching activity? In: *Agrarian Economy and Rural Development - Realities and Perspectives for Romania. 6th Edition of the International Symposium, November 2015*. ; 2015:23-28. Accessed June 7, 2022. http://hdl.handle.net/10419/163274
5. USDA Foreign Agricultural Service. *Turkey Livestock Annual Report 2018*.; 2018.
6. Khatibi M, Abdulaliyev G, Azimov A, et al. Working towards development of a sustainable brucellosis control programme, the Azerbaijan example. *Res Vet Sci*. 2021;137:252-261. doi:10.1016/J.RVSC.2021.05.014
7. Bulut A, Goshen T, Baziladze V. Item 4b. Current FAST situation in Türkiye, Israel, Georgia. In: *45th General Session of the European Commission for the Control of Foot-and-Mouth Disease (EuFMD) 4-5 May 2023*. ; 2023:9-12. https://www.fao.org/3/cc7080en/cc7080en.pdf
8. Fairtrade. *Powering up Smallholder Farmers to Make Food Fair: A Five Point Agenda*.; 2013. Accessed July 6, 2022. https://www.fairtrade.net/library/powering-up-smallholder-farmers-to-make-food-fair-a-five-point-agenda
9. FAO. Pastoralism - Policy Support and Governance Gateway. Accessed July 11, 2022. https://www.fao.org/policy-support/policy-themes/pastoralism/en/
10. Morris ST. Overview of sheep production systems. *Advances in Sheep Welfare*. Published online January 1, 2017:19-35. doi:10.1016/B978-0-08-100718-1.00002-9
11. CDC. Principles of Epidemiology | Lesson 1 - Section 11. Deputy Director for Public Health Science and Surveillance, Center for Surveillance, Epidemiology, and Laboratory Services, Division of Scientific Education and Professional Development. Published 2012. Accessed July 13, 2022. https://www.cdc.gov/csels/dsepd/ss1978/lesson1/section11.html
12. Riley LW. Differentiating Epidemic from Endemic or Sporadic Infectious Disease Occurrence. *Microbiol Spectr*. 2019;7(4). doi:10.1128/MICROBIOLSPEC.AME-0007-2019
13. Cartín-Rojas A. Transboundary Animal Diseases and International Trade. *International Trade from Economic and Policy Perspective*. Published online August 22, 2012. doi:10.5772/48151
14. Domenech J, Lubroth J, Eddi C, Martin V, Roger F. Regional and international approaches on prevention and control of animal transboundary and emerging diseases. *Ann N Y Acad Sci*. 2006;1081:90-107. doi:10.1196/annals.1373.010
15. Alleweldt F, Upton M, Kara Ş, Beteille R. The cost of national prevention systems for animal diseases and zoonoses in developing and transition countries. *OIE Revue Scientifique et Technique*. Published online 2012. doi:10.20506/rst.31.2.2145
